# Axillary silicone lymphadenopathy secondary to augmentation mammaplasty

**DOI:** 10.4103/0970-0358.73453

**Published:** 2010

**Authors:** Dimitrios M. Dragoumis, Anthoula S. Assimaki, Triantafyllos I. Vrizas, Aris P. Tsiftsoglou

**Affiliations:** Department of General Surgery, Breast Division, St Luke’s Hospital, Panorama, 55 236, Thessaloniki, Greece; 1 Department of Plastic Surgery, St Luke’s Hospital, Panorama, 55 236, Thessaloniki, Greece

**Keywords:** Augmentation, breast prostheses, implant, mammaplasty, silicone lymphadenopathy, ruptured breast implant

## Abstract

We report a case involving a 45-year-old woman, who presented with an axillary mass 10 years after bilateral cosmetic augmentation mammaplasty. A lump was detected in the left axilla, and subsequent mammography and magnetic resonance imaging demonstrated intracapsular rupture of the left breast prosthesis. An excisional biopsy of the left axillary lesion and replacement of the ruptured implant was performed. Histological analysis showed that the axillary lump was lymph nodes containing large amounts of silicone. Silicone lymphadenopathy is an obscure complication of procedures involving the use of silicone. It is thought to occur following the transit of silicone droplets from breast implants to lymph nodes by macrophages and should always be considered as a differential diagnosis in patients in whom silicone prostheses are present.

## INTRODUCTION

During the last four decades, silicone has become one of the most extensively utilized materials for the manufacture of breast implants, mainly because it is non-biodegradable and elicits no or little reaction from human tissue. This wide application of implanted silicone prostheses stems from their biological stability, the long-term preservation of their physical properties, combined with minimal tissue reaction and lack of immunogenicity. In spite of that reputation, side effects associated with the utilization of silicone have been well documented in literature. One uncommon side effect of mammary augmentation is silicone lymphadenopathy, defined as the presence of silicone in a lymph node.[1] This case report describes this obscure complication of silicone breast implantation and discusses thoroughly the challenging diagnostic and therapeutic implications of this clinical enigma.

## CASE REPORT

A 45-year-old woman presented to our clinic complaining of a lump, located in the left axilla. Despite having been aware of this lesion for two months, she had not sought immediate medical treatment, until she began to notice intermittent pain in her left axilla and a sensation of heaviness. She had undergone bilateral breast augmentation, using subglandular cohesive gel silicone implants of textured shell surface 10 years ago (Mentor™ – 220 cc each).

On physical examination, there was a relatively mobile, hard and non-tender mass, approximately 3cm in diameter that was located in the left axilla.

**Figure 1:**
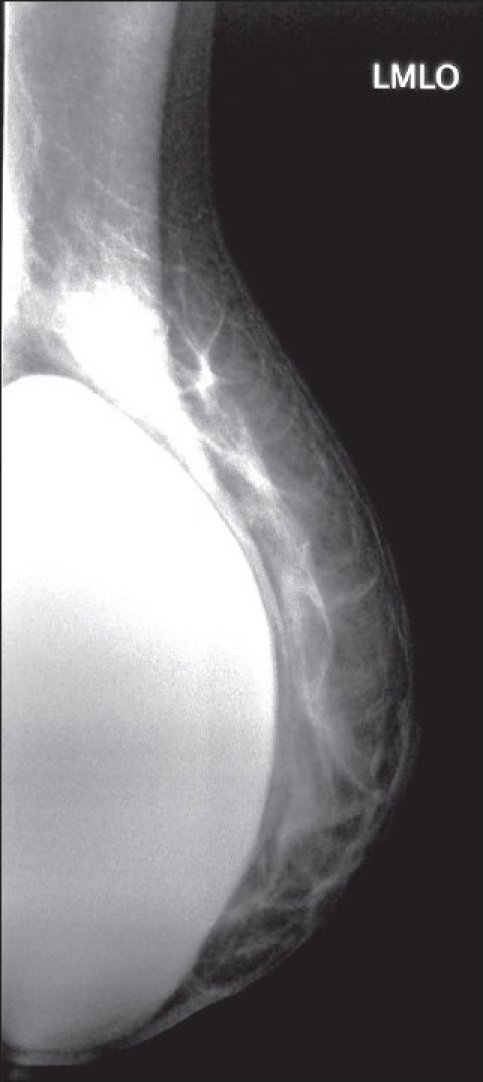
Mammography showing irregularity of the contour of the left breast implant and a radiodense mass in the left axilla

**Figure 2:**
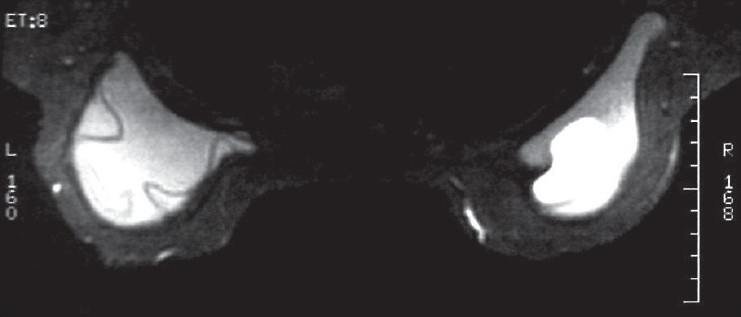
Axial magnetic resonance mammography revealing gross disorganization and collapse of the left implant with a positive ‘linguine sign’

**Figure 3:**
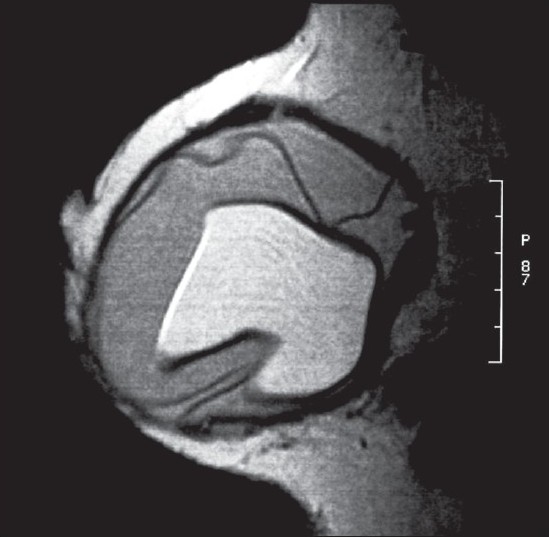
Sagittal magnetic resonance mammography demonstrating the collapsed intracapsular rupture of the left implant

Mammography demonstrated an irregular contour of the left implant and a highly radiodense axillary lesion, which corresponded to the palpable mass [Figure 1], while a subsequent breast magnetic resonance imaging (MRI) documented the intracapsular rupture (linguini sign) of the left breast prosthesis, but did not show evidence of silicone leakage from the implants [Figures 2 and 3]. Because the patient denied fine needle aspiration cytology (FNAC), excisional biopsy and frozen section analysis of the mass was proposed in order to confirm the benign nature of the lump. Before the excisional biopsy, the patient was reviewed as an outpatient by the plastic surgeon, who had performed the original augmentation procedure. A combined procedure involving excision biopsy of the left axillary lesion and replacement of the ruptured implant was eventually performed.

**Figure 4:**
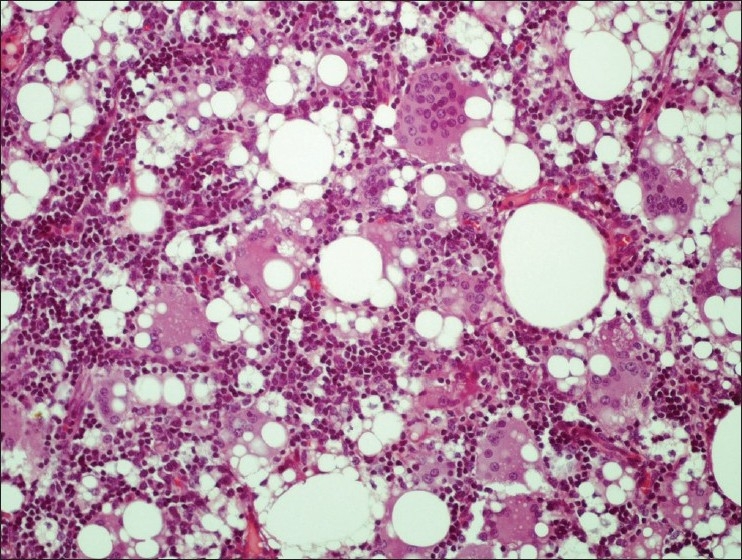
Histological examination showing lymph node with multinucleated giant cells, vacuoles and refractive material consistent with silicone (Haematoxylin and Eosin staining ×200)

**Figure 5:**
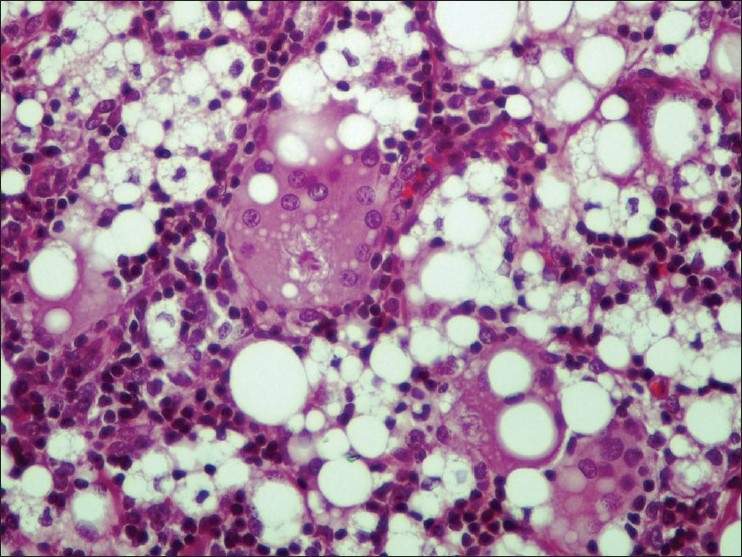
Higher magnifi cation photomicrograph revealing liquid silicone droplets appearing as round vacuoles of varying sizes in lymph node parenchyma (Haematoxylin and Eosin staining ×400)

On gross examination, small amount of pus-like fluid was seen to surround the ruptured implant. Four enlarged lymph nodes were abundant of clear viscous material, which oozed from the cut surface of the specimen. Subsequent histological analysis identified a histiocytic infiltrate with multinucleated giant cells, vacuoles and refractive material consistent with silicone lymphadenopathy [Figures 4 and 5].

Follow-up is satisfactory to date and 2 months later she remains well, with complete resolution of her initial postoperative discomfort.

## DISCUSSION

Silicone has been used in surgery for over 40 years in breast augmentation. It is composed of dimethylsiloxane polymers, which can result in differing properties according to the variation in their chain lengths and cross-links. Despite its initial reputation as a biologically inert material, it has been related with numerous complications including local and systemic granulomatous inflammatory reactions affecting breast tissue, lymph nodes, joint capsules, heart, liver, and kidneys. Silicone lymphadenopathy involving axillary lymph nodes is an uncommon complication of augmentation mammaplasty.[2–4]

Silicone particles can migrate through tissues by two distinct mechanisms. The first, following rupture or erosion of a silicone-containing surface and secondly, through continued leakage through an intact surface. The risk of rupture or leakage increases with increasing age of the implant, the site of implantation (retroglandular), the presence of local tissue contractures or symptoms and the type of implant. The average age at rupture varies between studies, but is in the region of 10 to 13 years and it is best diagnosed by MRI. Rupture is usually a harmless complication, which only rarely progresses and becomes symptomatic. When leakage does happen, silicone can cause fibrosis and foreign body reaction, especially when combined with certain fatty acids, resulting in pain and contractures. Once silicone particles have breached the confines of their prosthesis, they may be dispersed through any fibrotic reaction to regional lymph nodes by macrophages in the reticuloendothelial system. The granulomatous reactions may present as lymphadenopathy and, when present in the axilla, malignancy of the ipsilateral breast needs to be excluded.[2,5,6]

The presence of silicone droplets in lymph nodes of patients with breast implants suggests that the transit of various elements, either synthetic or biologic, from breast tissue to lymph nodes via lymphatic channels may have a significant passive component. This passive component may be a crucial determinant in the metastatic process. Silicone migration from breast implants to lymph nodes may therefore represent a model that could be useful in understanding the passive component of metastasis in breast cancer.[7]

The clinical importance of silicone lymphadenopathy has several different facets. In patients who have had post-mastectomy reconstructive surgery using silicone gel breast implants, the differential diagnosis of regional lymph node enlargement should include metastatic breast cancer, as well as silicone lymphadenopathy. In most individuals, who have had cosmetic surgery for breast augmentation, one must also recognize the potential for adverse health effects of silicone migration to regional lymph nodes. The association between silicone breast prostheses and systemic diseases is a highly controversial issue. Till now, most epidemiologic studies, found no association between breast implants and a variety of connective tissue diseases, despite the fact that Brown *et al*. have published a statistically significant link between ruptured silicone gel implants and fibromyalgia, as well as other autoimmune diseases.[8] On the other hand, there are numerous reports of symptoms in women with breast implants, including myalgia, arthralgia, fatigue and sleep disorders, but there is no adequate evidence of such a relation in the literature. Furthermore, the role of silicone in the development of lymphoma deserves mention, since there are several case reports describing primary breast lymphoma in patients with silicone gel breast implants, as well as patients with coexistent silicone lymphadenopathy and lymphoma in the same lymph node.[6,7,9]

In conclusion, silicone lymphadenopathy is a rare complication of procedures involving insertion of silicone-containing prostheses. This case study highlights the fact that patients need a thorough preoperative evaluation with histologic confirmation of the non-malignant nature of regional lymphadenopathy and reinforces the need to employ a high index of clinical suspicion, in order to exclude malignancy, without leading patients to dangerous overtreatment regimes.
